# The ApoA-IV–LRP1 Signaling Axis: A Novel Insulin-Independent Pathway for the Suppression of Diabetic Hyperglucagonemia

**DOI:** 10.3390/cells15131229

**Published:** 2026-07-07

**Authors:** Min Liu, Xenia Davis, Chih-Wei Ko, Ling Shen, Maureen Fitzgerald, Chunmin C. Lo, Patrick Tso

**Affiliations:** 1 Department of Pathology and Laboratory Medicine, University of Cincinnati College of Medicine, Cincinnati, OH 45237, USA; shenln@ucmail.uc.edu (L.S.); tsopp@ucmail.uc.edu (P.T.); 2 Department of Anesthesiology, Vanderbilt University Medical Center, Nashville, TN 37232, USA; xenia.d.davis@vumc.org; 3 Chroma Medicine, 201 Brookline Ave, Suite 1101, Boston, MA 02215, USA; ckbio87@gmail.com; 4 Department of Pharmacology, Physiology, & Neurobiology, University of Cincinnati College of Medicine, Cincinnati, OH 45237, USA; fitzgeme@ucmail.uc.edu; 5 Department of Biomedical Sciences and Diabetes Institute, Heritage College of Osteopathic Medicine, Ohio University, Athens, OH 45701, USA; loc1@ohio.edu

**Keywords:** Apolipoprotein A-IV, diet-induced obesity, streptozotocin, glucagon, LRP1, gluconeogenesis, insulin resistance, energy expenditure

## Abstract

**Highlights:**

**What are the main findings?**

- ApoA-IV prevents diet-induced obesity by increasing energy expenditure and shifting metabolic substrate preference toward lipid oxidation.
- ApoA-IV directly inhibits pancreatic α-cell glucagon secretion via an LRP1-dependent pathway, significantly attenuating hyperglycemia following severe β-cell loss.

**What are the implications of the main findings?**

- The ApoA-IV-LRP1 axis represents a novel therapeutic target for managing hyperglucagonemia and hepatic glucose production in diabetes.
- Enhancing ApoA-IV activity may provide a dual-action approach to treat metabolic syndrome by simultaneously reducing adiposity and improving glycemic regulation.

**Abstract:**

Apolipoprotein A-IV (ApoA-IV) is a glycoprotein secreted by the small intestine to regulate lipid metabolism and satiety. Its role in insulin-independent glucose homeostasis remains largely unknown. In this study, we demonstrate that intestinal ApoA-IV overexpression significantly attenuates diet-induced obesity and hyperglycemia following severe β-cell loss. Over a 20-week high-fat diet challenge, ApoA-IV transgenic (ApoA-IV-Tg) mice maintained significantly lower adiposity than wild-type controls, driven by elevated energy expenditure and fatty acid oxidation rather than reduced caloric intake. Beyond weight maintenance, ApoA-IV maintained excellent systemic glycemic control and enhanced peripheral insulin sensitivity. Most notably, ApoA-IV significantly attenuated hyperglycemia following streptozotocin (STZ)-induced β-cell ablation, maintaining glucose stability despite severe insulin deficiency. Mechanistically, this protection results from a blunted glucagon response and the subsequent suppression of the hepatic pCREB-G6Pase gluconeogenic signaling pathway. In vitro evidence confirms that ApoA-IV directly inhibits pancreatic α-cell glucagon secretion through an LDL receptor-related protein 1 (LRP1)-dependent pathway, reinforced by the precise co-localization of LRP1 and glucagon in pancreatic islets. Furthermore, ApoA-IV-Tg mice were protected from the STZ-induced corticosterone surge and systemic lipolysis. Collectively, these findings establish the ApoA-IV–LRP1 signaling axis as a potent metabolic switch, providing a promising insulin-independent strategy for managing obesity and diabetes.

## 1. Introduction

The global escalation of obesity and type 2 diabetes represents one of the most formidable challenges to modern public health, driven largely by chronic overnutrition and sedentary lifestyles [1,2]. Central to the pathogenesis of these metabolic disorders is the breakdown of energy homeostasis, characterized by excessive adiposity, systemic insulin resistance, and the progressive failure of pancreatic β-cells [3]. While current therapeutic strategies primarily focus on augmenting insulin secretion or sensitivity, there is increasing recognition that dysregulation of counterregulatory hormones, most notably hyperglucagonemia originating from pancreatic α-cells, plays a pivotal role in driving fasting hyperglycemia and metabolic dysfunction [4,5].

Apolipoprotein A-IV (ApoA-IV) is primarily synthesized by the small intestine, where its expression is acutely up-regulated in response to lipid absorption [6]. We previously reported that ApoA-IV knockout (KO) mice exhibit significant glucose intolerance and impaired insulin secretion in response to a glucose challenge, suggesting ApoA-IV as a necessary component of normal glycemic control [7]. Furthermore, exogenous ApoA-IV administration restores insulin secretion and improves glucose tolerance in ApoA-IV-KO mice [7]. Beyond its role in glucose-stimulated insulin secretion, ApoA-IV also acts as a potent satiety signal and a facilitator of systemic lipid metabolism [8]. Despite these established postprandial roles, the potential of ApoA-IV to regulate glucose levels through insulin-independent pathways, specifically by modulating the α-cell-to-liver axis, remains poorly defined.

Previously, we have demonstrated that ApoA-IV interacts with LDL receptor-related protein 1 (LRP1), the largest member of the LDL receptor family, within adipose tissue [9]. LRP1 is a versatile endocytic receptor highly expressed in key metabolic tissues, including the liver and the pancreas, where it serves as a molecular “switch” for various signaling ligands [10,11]. Given that LRP1 influences both cellular lipid uptake and hormonal signaling, we hypothesized that the ApoA-IV-LRP1 interaction might represent a novel and important regulatory mechanism to suppress glucagon-driven gluconeogenesis.

In the present study, we utilized an ApoA-IV-transgenic (ApoA-IV-Tg) mouse model to investigate the impact of chronic ApoA-IV overexpression on diet-induced obesity and glucose homeostasis. This transgenic mouse line, which carries several copies of the mouse *ApoA4* gene, exhibits a 2- to 3-fold elevation of ApoA-IV expression specifically within intestinal enterocytes [12]. Initial characterization revealed that ApoA-IV-Tg mice fed an atherogenic diet exhibit significantly elevated HDL levels and reduced levels of unesterified cholesterol. This favorable lipid profile confers robust protection against atherosclerosis by shielding the mice from diet-induced aortic lesions [12]. In the present study, we demonstrate that ApoA-IV overexpression provides robust protection against adiposity by increasing energy expenditure and fatty acid oxidation. Furthermore, using a streptozotocin (STZ)-induced model of β-cell ablation, we show that ApoA-IV maintains relatively normal glycemic control even in the absence of insulin. This protection is mediated by a direct, LRP1-dependent inhibition of α-cell glucagon secretion, thereby suppressing the hepatic glucagon-CREB-G6Pase signaling pathway. These findings reveal a previously unrecognized role for ApoA-IV as a potent regulator of α-cell function, providing a new conceptual framework for the treatment of both Type 1 and Type 2 diabetes.

## 2. Materials and Methods

### 2.1. Animal Models and Dietary Intervention

The ApoA-IV-transgenic (ApoA-IV-Tg) mouse colony was generously provided by Dr. Karen Reue of UCLA, whose team previously demonstrated a significant increase in ApoA4 gene expression, specifically in the intestine [12]. The colony was maintained on a C57BL/6J background through more than 10 generations of back-crossing. To validate the expression status of our colony, we confirmed approximately a 3-fold increase in ApoA4 mRNA levels in the intestines of chow-fed ApoA-IV-Tg mice compared to wild-type (WT) controls.

Male mice were housed in an AAALAC-accredited facility at the University of Cincinnati under a controlled 12 h light/dark cycle (06:00–18:00) and at 22 °C with *ad libitum* access to food and water. Following genotype confirmation via PCR, 10-to-14-week-old mice were transitioned to a high-fat diet (HFD, Diet D03082706, Research Diets, New Brunswick, NJ, USA), containing 20% fat by weight, providing 40% of total calories (4.54 kcal/g) [13], for a 20-week experimental period. Throughout the experimental period, body weight and caloric intake were recorded weekly. Blood glucose was measured biweekly from tail vein samples after a 5 h fast. Upon completion of the study, mice were euthanized for blood and tissue collection. All procedures were performed in strict accordance with protocols (21-08-09-01 and 24-10-14-01) approved by the University of Cincinnati Institutional Animal Care and Use Committee (IACUC).

### 2.2. Reagents and Antibodies

STZ was purchased from Sigma-Aldrich (St. Louis, MO, USA). The ELISA kit for mouse insulin was purchased from EMD Millipore Corp. (Billerica, MA, USA). Glucagon and corticosterone (CORT) levels were measured using kits from Crystal Chem, Inc. (Elk Grove Village, IL, USA) and Enzo Life Sciences, Inc. (Ann Arbor, MI, USA), respectively. Circulating triglycerides (TG) and total cholesterol were quantified using a Randox assay kit (Randox Laboratories Ltd., Kearneysville, WV, USA) and the Infinity cholesterol reagent (Thermo Fisher Scientific, Waltham, MA, USA). Phospholipid concentrations were determined using specialized enzymatic kits (Phospholipids C, Wako Life Sciences, Inc., Mountain View, CA, USA). Free glycerol and free fatty acids were measured using reagents from Sigma-Aldrich (St. Louis, MO, USA) and Fujifilm Wako Pure Chemical Co. (Osaka, Japan), respectively. The antibodies for phosphorylated cAMP-response-element-binding protein (pCREB, Cat# 9198) and total CREB (Cat# 9197) were from Cell Signaling Technology, Inc. (Danvers, MA, USA). Primary antibodies used for immunohistochemistry included rabbit anti-LRP1 (Affinity Biosciences, Cincinnati, OH, USA, Cat# DF2935) and mouse anti-glucagon (Sigma-Aldrich, Cat# G2645).

### 2.3. Body Composition

Body composition was analyzed in vivo using an EchoMRI-100 system (Echo Medical Systems, Houston, TX, USA) [14]. The system quantified whole-body fat, lean mass, and water content without the need for sedation. Adiposity and lean mass indices were subsequently calculated as fractions of total body weight and reported as percentages.

### 2.4. Glucose Tolerance Assay

To evaluate systemic glucose homeostasis, intraperitoneal glucose tolerance tests (IPGTT) were performed on HFD-fed cohorts at 0, 4, and 16 weeks of dietary intervention [14]. Following a 5 h fast, animals received an i.p. bolus of glucose (2 g/kg body weight). Glycemic profiles were monitored via tail vein sampling at baseline (0 min) and at 15, 30, 60, and 120 min post-injection using a handheld glucometer (Freestyle Lite; Abbott, Chicago, IL, USA). Concurrently, plasma insulin concentrations were quantified using a commercial Mouse insulin ELISA kit, according to the manufacturer’s instructions.

### 2.5. Analysis of Indirect Calorimetry and Physical Activity

Metabolic parameters, including oxygen consumption (VO_2_) and carbon dioxide production (VCO_2_), were quantified using an indirect calorimetry system (Oxymax v. 5.35; Columbus Instruments, Columbus, OH, USA) [14]. Mice were individually housed in metabolic chambers within the Comprehensive Laboratory Animal Monitoring System (CLAMS) and provided a 24 h acclimation period. Measurements were recorded continuously for 24 h with *ad libitum* access to food and water. The Respiratory Exchange Ratio (RER) was calculated as the ratio of VCO_2_ to VO_2_. Energy expenditure (EE) was determined using the following formula: Heat = (3.941 + 1.106 × RER) × VO_2_. Data are expressed as kcal/h/kg of body weight. Concurrent physical activity was monitored via infrared beam interruptions (Smart Frame; Hamilton-Kinder, Poway, CA, USA). Locomotor activity was integrated every 60 min, with total 24 h ambulatory counts calculated as the sum of all x-axis beam breaks [14].

### 2.6. Measurement of Plasma Lipid Contents

Plasma lipid profiles were determined using samples obtained from mice following a 5 h fast. Circulating triglycerides, total cholesterol, and phospholipids were quantified using the respective enzymatic kits and reagents described above [14]. All biochemical assays were performed in strict accordance with the manufacturers’ instructions.

### 2.7. HOMA-IR Calculation

The Homeostatic Model Assessment of Insulin Resistance (HOMA-IR) was calculated to assess insulin sensitivity in the WT and ApoA-IV-Tg mice. The assessment was derived from 5 h fasting blood glucose and plasma insulin concentrations using the following formula: HOMA-IR = [Fasting Glucose (mg/dL) × Fasting Insulin (mU/L)]/405 [15].

### 2.8. STZ-Treatment

Mice were fasted overnight and then received a single ip injection of a freshly prepared solution of STZ (200 mg/kg), a selective pancreatic β-cell toxin, dissolved in 50 mM citrate buffer (pH 4.5). Control mice received an equivalent volume of citrate buffer alone. A 10% sucrose solution was provided for the first 24 h after STZ treatment. During the experiment, the mice were fasted daily from 0900 to 1400 h prior to blood withdrawal for glucose and insulin determination. Daily food intake and body weight were monitored after injection. Mice were euthanized on Day 5 post-STZ treatment following a 5 h fast; blood and liver were subsequently harvested for analysis.

### 2.9. Measurements of Plasma Samples

Plasma collected from the trunk blood of STZ-treated mice was used to measure glucagon, corticosterone (CORT), free glycerol, and free fatty acids using commercial reagents according to the manufacturers’ instructions.

### 2.10. Western Blot for Target Protein Measurements

Liver samples were homogenized, and the supernatant (containing 10 µg of protein) was loaded onto 4–15% polyacrylamide gradient gels, transferred to polyvinylidene difluoride membranes, and blotted with a pCREB antibody (1:1000 dilution) overnight at 4 °C. After rinsing with TBS with 0.1% Tween 20, membranes were incubated with peroxidase-conjugated anti-rabbit secondary antibody at 1:10,000 for 60 min. Blots were developed using enhanced chemiluminescence reagents (Bio-Rad Laboratories Inc., Hercules, CA, USA). Images were captured by the ChemiDoc System, and band intensities were quantified using Image Lab software (version 6.0.1, Bio-Rad Laboratories, Inc.). The blots were stripped and re-incubated with an antibody against CREB (1:1000 dilution). The amount of pCREB protein was normalized to the respective individual density values reflecting total CREB protein levels and was expressed as a ratio.

### 2.11. qPCR for G6Pase and PEPCK mRNA Measurement

Total RNA was extracted from the liver using a PureLink RNA Mini Kit (Thermo Fisher Scientific, Waltham, MA, USA). RNA was quantified using a NanoDrop 2000 (Thermo Fisher Scientific). 250 ng of RNA from each sample was used for reverse transcription to cDNA with a Transcriptor First Strand cDNA Synthesis Kit (Roche Diagnostics Corporation, Indianapolis, IN, USA). Glucose-6-phosphatase (G6pase) and phosphoenolpyruvate carboxykinase (PEPCK) mRNA levels were quantified by quantitative real-time PCR (qPCR) using TaqMan Master Mix with TaqMan Gene Expression Assay for PEPCK and G6pase with a StepOne™ Plus device (Thermo Fisher Scientific). Relative mRNA expression levels were calculated using the comparative 2^−ΔΔCT^ algorithm. 18S mRNA levels from each sample were used as the internal control reference gene to normalize the transcript levels. The stability of 18S rRNA expression was evaluated across all experimental groups, showing no statistically significant variation in raw threshold cycle (*C_T_*) values between genotypes or treatment conditions (*p* < 0.05) thereby confirming its suitability as a stable reference.

### 2.12. Pancreatic Islet Culture and Ex Vivo Glucose-Stimulated Glucagon Secretion (GSGS) Assay

Pancreatic islets were isolated using the collagenase P digestion method. Briefly, the common bile duct was cannulated and perfused with 3 mL of ice-cold Krebs-Ringer bicarbonate (KRB) buffer containing 1.0 mg/mL collagenase P (Roche Diagnostics). The pancreas was surgically excised and incubated in a water bath at 37 °C for exactly 16 min to achieve optimal digestion. Digestion was quenched with ice-cold KRB buffer containing 10% FBS, and islets were separated using a Histopaque density gradient followed by manual hand-picking. Islets were cultured in RPMI 1640 supplemented with 10% (*v*/*v*) FBS and 11 mM glucose at 37 °C for 24 h, as previously described [7].

For the secretion assay, size-matched islets (15 per well in a 24-well plate) were equilibrated at 37 °C for 1 h in KRB buffer containing 11 mM glucose. To investigate the role of LRP1 signaling, receptor-associated protein (RAP, 0.3 μM), an LRP1 antagonist, was added to designated wells 15 min before the end of the equilibration phase.

Following equilibration, islets were transferred to KRB buffer containing 2 mM glucose to stimulate glucagon secretion and incubated for an additional 1 h in the presence or absence of ApoA-IV (2.5, 5, and 10 μg/mL) and RAP (0.3 μM). At the end of this 60 min stimulation period, supernatants were carefully collected into pre-chilled microcentrifuge tubes and centrifuged at 1000× *g* for 5 min at 4 °C to remove any remaining islets or debris. Glucagon concentrations in the medium were subsequently quantified using a commercial ELISA kit.

### 2.13. Fluorescence Immunohistochemistry

Pancreatic tissues harvested from WT mice were fixed in 10% neutral buffered formalin (pH 7.0) for 48 h at 4 °C. Following paraffin embedding, 5-μm sections were deparaffinized in xylene and rehydrated through a graded series of ethanol, as described previously [9]. For the co-localization of LRP1 and glucagon, sections were incubated for 1 h in blocking buffer, then incubated overnight at 4 °C with a mixture of primary antibodies: rabbit anti-LRP1 (1:50) and mouse anti-glucagon (1:1000), prepared in blocking buffer. After washing, the sections were incubated for 45 min at room temperature with the following fluorophore-conjugated secondary antibodies: Cy3-conjugated goat anti-rabbit (for LRP1) and Alexa Fluor 488-conjugated goat anti-mouse (for glucagon). Upon completion of fluorophore incubations and washing, the sections were incubated in DAPI (Invitrogen-Cat # 62248) diluted 1:1000 in 0.1 M PBS for 10 min, washed, and cover-slipped using gelvatol mounting media. Fluorescent images were acquired using a Zeiss Axio Imager 2 microscope equipped with an ApoTome.2 optical sectioning system (Carl Zeiss, Thornwood, NY, USA) at 20× or 40× magnification. Digital processing, including pseudocoloring and image merging, was performed using Axiovision software (version 4.8, Carl Zeiss) [9].

### 2.14. Statistical Analysis

Data are expressed as mean ± standard error of the mean (SEM). All in vivo data are representative of *n* = 5–8 biological replicates (individual mice) per group, and in vitro islet assays represent a minimum of *n* = 4 independent biological experiments, with each condition run in technical triplicate. Statistical analyses and graphical representations were performed using GraphPad Prism 10.0 (GraphPad Software, La Jolla, CA, USA). Prior to selecting the final statistical test, all datasets were formally verified for normality using the Shapiro–Wilk test and for homogeneity of variances using the Brown–Forsythe or Bartlett’s test. Differences between genotypes (WT and ApoA-IV-Tg) and experimental conditions were evaluated using two-way analysis of variance (ANOVA) followed by Sidak’s post hoc test for multiple comparisons. For comparisons involving a single independent variable, a one-way ANOVA followed by Tukey’s post hoc test or an unpaired Student’s *t*-test was utilized as appropriate. Statistical significance was defined as *p* < 0.05.

## 3. Results

### 3.1. ApoA-IV Overexpression Attenuates Diet-Induced Obesity and Adiposity

**Figure 1:**
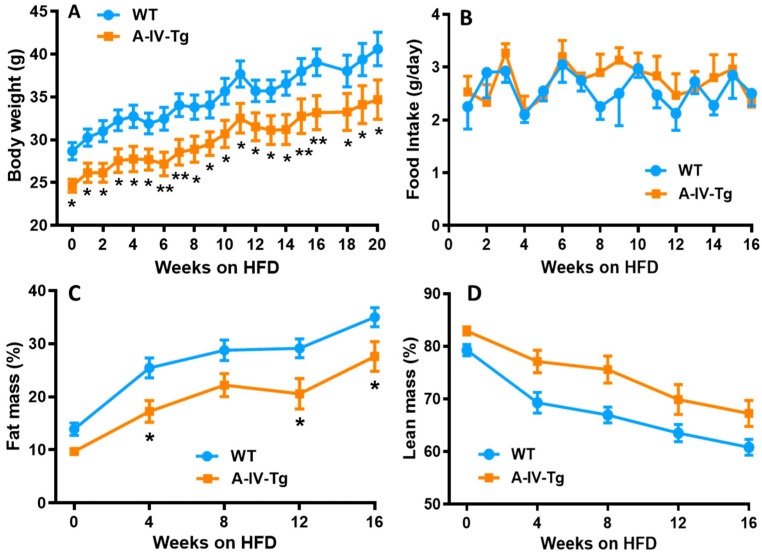
Resistance to diet-induced obesity in ApoA-IV-Tg mice during HFD feeding. (**A**) Absolute body weight and (**B**) food intake in wild-type (WT) and ApoA-IV-Tg (A-IV-Tg) mice over a 16-week HFD challenge. Body composition, including fat mass (**C**) and lean mass (**D**), was assessed via EchoMRI every 4 weeks over 16 weeks of HFD feeding. Data are presented as mean ± SEM (*n* = 6–8 per group). * *p* < 0.05, ** *p* < 0.01, vs. WT controls at the corresponding time point.

To determine the physiological impact of ApoA-IV on energy balance, we monitored body weight, food intake, and body composition in WT and ApoA-IV-Tg mice during an HFD challenge. At the start of the study (Week 0), ApoA-IV-Tg mice had a significantly lower baseline body weight than WT controls. Throughout the study, both groups showed a progressive increase in body weight, but ApoA-IV-Tg mice maintained significantly lower absolute body weight from baseline through week 20 (Figure 1A). Concurrently, the total body weight gain over the 20-week challenge was significantly lower in the transgenic group than in WT controls (9.7 ± 0.7 g vs. 12.1 ± 0.9 g; *p* < 0.05), confirming active resistance to weight gain. Interestingly, this resistance to weight gain in ApoA-IV-Tg mice occurred independently of changes in appetite, as daily food intake remained comparable between the two genotypes throughout the study (Figure 1B).

Further longitudinal assessment of body composition using EchoMRI revealed that the reduced body weight in ApoA-IV-Tg mice was specifically characterized by lower adiposity. ApoA-IV-Tg mice maintained a significantly lower body fat percentage compared to WT mice at weeks 4, 12, and 16 (Figure 1C). While the percentage of lean mass was higher in ApoA-IV-Tg mice than in WT controls, this difference did not reach statistical significance (Figure 1D). These data suggest that intestinal ApoA-IV overexpression confers significant resistance to HFD-induced obesity, primarily by limiting fat accumulation, without detrimental effects on the preservation of lean tissue.

Consistent with this, ApoA-IV-Tg mice showed a trend toward reduced adiposity, characterized by lower percentages of visceral fat (Supplementary Figure S1A) and subcutaneous fat (Supplementary Figure S1B) than WT controls. While white adipose tissue decreased, a modest increase was observed in brown adipose tissue (BAT) levels in the ApoA-IV-Tg group (Supplementary Figure S1C), suggesting a potential shift in metabolic activity or thermogenesis. Additionally, liver weight was slightly lower in ApoA-IV-Tg mice relative to the WT group (Supplementary Figure S1D). Taken together, these data suggest that ApoA-IV overexpression may provide a protective effect against fat accumulation in both peripheral and visceral compartments.

### 3.2. Elevated Energy Expenditure and Enhanced Lipid Utilization in ApoA-IV-Tg Mice

**Figure 2:**
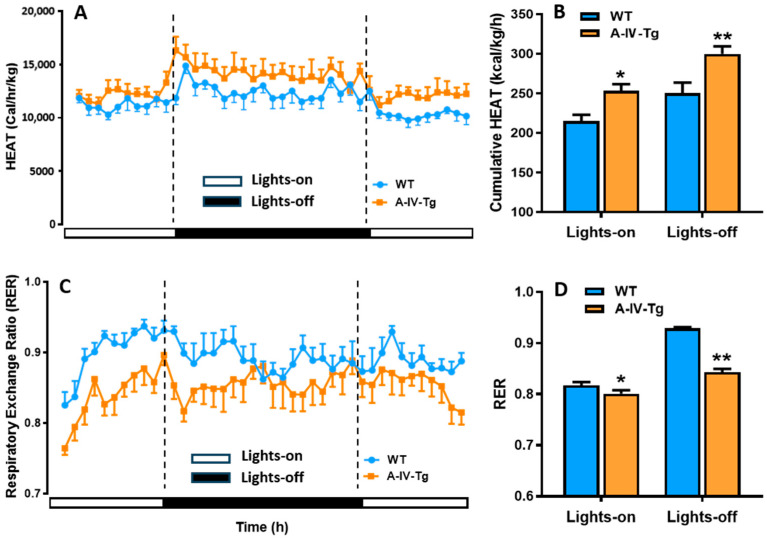
Energy expenditure in WT and apoA-IV-Tg mice during HFD feeding. (**A**) Indirect calorimetry measurements of energy expenditure (HEAT) at week 17. (**A**,**B**) ApoA-IV-Tg mice exhibited significantly elevated HEAT compared to WT mice, particularly during the transition to the dark phase. (**C**,**D**) Respiratory Exchange Ratio (RER) was significantly lower in ApoA-IV-Tg mice during both light and dark phases, indicating a shift toward fatty acid oxidation. In the calorimetry plot, the white and black bars on the x-axis represent the light and dark cycles, respectively. Data are presented as mean ± SEM (*n* = 5–6 per group). * *p* < 0.05, ** *p* < 0.01, vs. WT controls.

To determine if the metabolic advantages of ApoA-IV overexpression persisted throughout chronic overnutrition, energy expenditure and substrate utilization were evaluated at week 17 of the HFD challenge. ApoA-IV-Tg mice exhibited significantly elevated energy expenditure (HEAT) compared to WT controls. This was particularly evident during the transition to the dark phase, with the transgenic group showing significantly higher cumulative HEAT during the light and dark cycles (Figure 2A,B). This elevation in thermogenesis was mirrored by considerably higher rates of oxygen consumption and carbon dioxide production in the transgenic group (Supplemental Figure S2).

Furthermore, a significant divergence in the respiratory exchange ratio (RER) was observed by week 17. ApoA-IV-Tg mice exhibited a significantly lower RER during the light and dark phases compared to WT mice (Figure 2C,D). This shift toward a lower RER indicates that the transgenic mice maintained a greater reliance on fatty acid oxidation for energy, even under the pressure of prolonged HFD feeding. Importantly, these metabolic changes occurred in the absence of alterations in physical movement, as locomotor activity remained comparable between WT and ApoA-IV-Tg mice across both light and dark cycles (Supplementary Figure S3). Collectively, these results suggest that chronic ApoA-IV overexpression promotes a sustained increase in metabolic rate and facilitates more efficient lipid utilization independent of physical activity.

### 3.3. Improved Plasma Lipid Profile in ApoA-IV-Tg Mice

**Figure 3:**
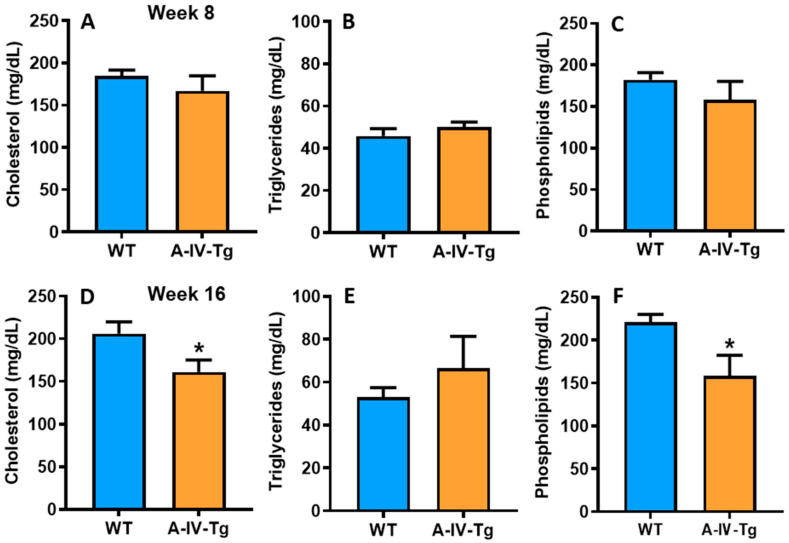
Plasma lipid profiles in WT and apoA-IV-Tg mice during HFD feeding. Plasma levels of cholesterol (**A**,**D**), triglycerides (**B**,**E**), and phospholipids (**C**,**F**) were measured at weeks 8 and 16 of HFD feeding. While lipid profiles were comparable in week 8, ApoA-IV-Tg mice exhibited significantly lower circulating cholesterol and phospholipid levels than WT controls by week 16. Data are presented as mean ± SEM (*n* = 6–8 per group). * *p* < 0.05 vs. WT mice.

To assess the impact of ApoA-IV overexpression on systemic lipid metabolism during chronic feeding of an HFD, plasma lipid profiles were analyzed at Weeks 8 and 16 of the HFD challenge. At Week 8, no significant differences were observed between WT and ApoA-IV-Tg mice regarding circulating levels of cholesterol, triglycerides, or phospholipids (Figure 3A–C). However, by Week 16, a clear divergence in the lipid profile emerged. ApoA-IV-Tg mice exhibited significantly lower plasma cholesterol levels compared to WT controls (Figure 3D). Similarly, plasma phospholipid concentrations were significantly reduced in the ApoA-IV-Tg group relative to WT mice (Figure 3F). Triglyceride levels did not differ significantly between transgenic and WT mice at either Week 8 or Week 16 (Figure 3E). These results demonstrate that long-term overexpression of ApoA-IV helps maintain lower circulating cholesterol and phospholipid levels during prolonged HFD feeding, consistent with the observed protection against diet-induced obesity and enhanced metabolic efficiency.

### 3.4. ApoA-IV Overexpression Improves Glycemic Control and Insulin Sensitivity

**Figure 4:**
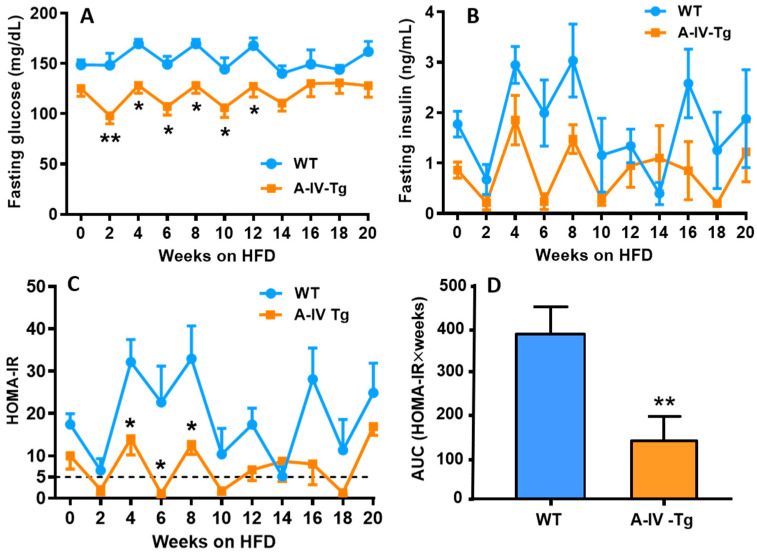
ApoA-IV overexpression attenuates the cumulative burden of insulin resistance. (**A**,**B**) Fasting blood glucose and plasma insulin levels were measured bi-weekly over 20 weeks of HFD feeding. (**C**) Longitudinal HOMA-IR scores calculated from fasting glucose and insulin values to evaluate systemic insulin sensitivity. (**D**) Total HOMA-IR AUC: Integration of the longitudinal HOMA-IR data from week 0 to week 20 using the trapezoidal rule. Data are expressed as mean ± SEM (*n* = 5–7 per group). * *p* < 0.05, ** *p* < 0.01 vs. WT mice.

To evaluate the impact of ApoA-IV on glucose homeostasis during chronic overnutrition, fasting glucose and insulin levels were monitored throughout a 20-week HFD challenge. ApoA-IV-Tg mice consistently maintained lower fasting blood glucose levels than WT controls, with statistical significance observed at multiple time points between weeks 2 and 12 (Figure 4A). Interestingly, this statistical divergence narrowed between weeks 14 and 20, suggesting that the fasting glycemic benefit may reach a plateau during prolonged, ultra-long-term high-fat feeding. Fasting insulin levels followed a similar downward trend in the transgenic group, suggesting a reduced insulin requirement to maintain glucose levels (Figure 4B).

A notable metabolic improvement was observed in HOMA-IR, with ApoA-IV-Tg mice exhibiting significantly lower scores at weeks 4 and 8 compared with WT mice (Figure 4C). To quantify the total integrated burden of insulin resistance throughout the 20-week intervention, the Area Under the Curve (AUC) for longitudinal HOMA-IR values was calculated. This analysis revealed a significant reduction in the cumulative HOMA-IR burden in ApoA-IV-Tg mice compared to WT controls (Figure 4D), indicating that ApoA-IV overexpression preserves insulin sensitivity and significantly mitigates the development of HFD-induced insulin resistance.

### 3.5. ApoA-IV Overexpression Enhances Systemic Glucose Tolerance and Reduces Insulin Requirement

**Figure 5:**
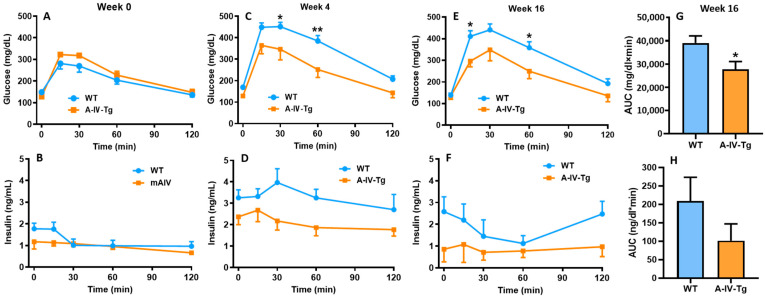
ApoA-IV overexpression improves glucose tolerance and reduces insulin demand during chronic HFD feeding. (**A**–**F**): IPGTT and corresponding plasma insulin profiles performed at Week 0 (**A**,**B**), Week 4 (**C**,**D**), and Week 16 (**E**,**F**) of HFD feeding in WT and ApoA-IV-Tg mice. (**G**,**H**): Integrated area under the curve (AUC) for blood glucose (**G**) and plasma insulin (**H**) at the 16-week marks. Data are presented as mean ± SEM (*n* = 6–7 per group). * *p* < 0.05; ** *p* < 0.01 vs. WT mice.

To further investigate the longitudinal impact of ApoA-IV on systemic glucose handling, intraperitoneal glucose tolerance tests (IPGTTs) were performed at baseline (Week 0), Week 4, and Week 16 during the HFD intervention. At baseline (Week 0), both WT and ApoA-IV-Tg mice exhibited similar glucose excursions and insulin responses (Figure 5A,B). However, after 4 weeks of HFD, ApoA-IV-Tg mice exhibited significantly improved glucose clearance compared with WT controls, with lower blood glucose levels at 30 and 60 min post-injection (Figure 5C). This enhanced glucose tolerance was maintained through Week 16, during which ApoA-IV-Tg mice continued to exhibit lower peak glucose levels (Figure 5E) and a significantly reduced total glucose AUC compared with WT mice (Figure 5G).

Notably, the improved glucose profiles in ApoA-IV-Tg mice were achieved despite lower circulating insulin levels during the challenge (Figure 5D,F), suggesting enhanced peripheral insulin sensitivity. While the insulin AUC at Week 16 showed a downward trend in the ApoA-IV-Tg group, this difference did not reach statistical significance (Figure 5H). Collectively, these findings indicate that ApoA-IV overexpression mitigates the development of glucose intolerance and reduces the compensatory insulin demand typically associated with prolonged high-fat feeding [16].

### 3.6. ApoA-IV Overexpression Attenuates Hyperglycemia Following Streptozotocin-Induced β-Cell Injury

**Figure 6:**
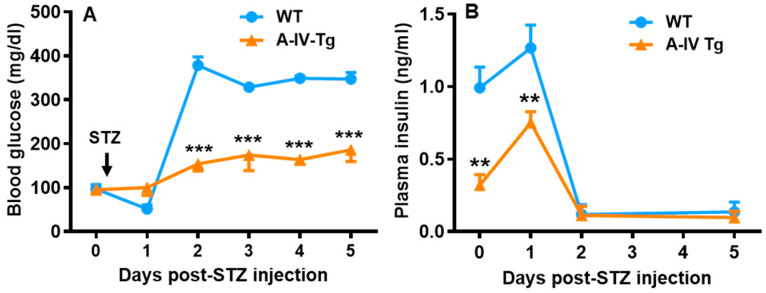
ApoA-IV overexpression attenuates STZ-induced hyperglycemia. (**A**) Fasting blood glucose levels were measured daily in WT and ApoA-IV-Tg mice following a single ip injection of STZ. While WT mice developed severe hyperglycemia by Day 2, ApoA-IV-Tg mice were significantly protected throughout the 5-day study period. (**B**) Plasma insulin concentrations were monitored during the STZ challenge. By Day 2, insulin levels in both groups had declined to near baseline levels. Data are presented as mean ± SEM (*n* = 5–8 per group). ** *p* < 0.01; *** *p* < 0.001 vs. WT mice.

To evaluate the glucose-lowering potential of ApoA-IV in a model of insulin deficiency, WT and ApoA-IV-Tg mice were subjected to a single high-dose injection of streptozotocin (STZ, 200 mg/kg). Prior to the challenge, both groups maintained similar fasting blood glucose levels; however, after STZ administration, WT mice developed rapid and severe hyperglycemia, with blood glucose exceeding 380 mg/dL by day 2 (Figure 6A). In contrast, ApoA-IV-Tg mice were significantly protected against STZ-induced hyperglycemia, maintaining blood glucose levels below 200 mg/dL throughout the 5-day observation period (*p* < 0.001 vs. WT mice).

While both genotypes showed a transient rise in insulin on Day 1 post-injection due to acute β-cell lysis, levels dropped precipitously by Day 2 and became nearly undetectable by Day 5, confirming successful β-cell ablation (Figure 6B). Notably, ApoA-IV-Tg mice exhibited significantly lower plasma insulin levels than WT controls at baseline (Day 0) and on Day 1. Despite a similar degree of severe insulin deficiency by the end of the study, ApoA-IV-Tg mice remained resistant to overt hyperglycemia, suggesting that the glucose-lowering effects of ApoA-IV in this model may be mediated through insulin-independent pathways or enhanced sensitivity to residual insulin.

Furthermore, following STZ treatment, both genotypes exhibited a rapid and progressive decline in body weight. However, ApoA-IV-Tg mice maintained significantly lower body weights than the WT group throughout the study (Supplementary Figure S4A). Notably, these differences in body weight were not attributable to alterations in appetite, as daily food intake remained comparable across all experimental groups (Supplementary Figure S4B).

### 3.7. ApoA-IV Overexpression Suppresses the Hepatic Glucagon-CREB-G6Pase Axis Post-STZ Treatment

**Figure 7:**
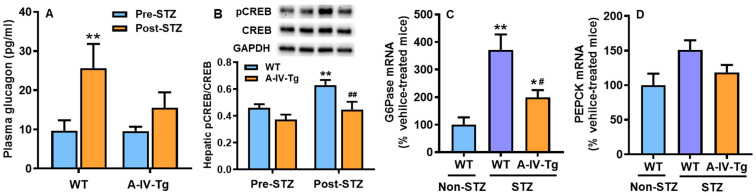
ApoA-IV overexpression attenuates STZ-induced hyperglucagonemia and hepatic gluconeogenic signaling. (**A**) Fasting plasma glucagon levels in WT and ApoA-IV-Tg mice before (Pre-STZ) and after (Post-STZ) STZ-induced β-cell ablation. (**B**) Representative Western blot and densitometric quantification of hepatic CREB phosphorylation (pCREB) normalized to total CREB. Hepatic mRNA expression of G6Pase (**C**) and PEPCK (**D**) measured via RT-qPCR. Expression levels are normalized to non-STZ WT controls. Data are expressed as mean ± SEM (*n* = 6–7 per group). Significance was determined by ANOVA: * *p* < 0.05; ** *p* < 0.01, vs. WT mice without STZ-treatment. ^#^ *p* < 0.05; ^##^ *p* < 0.01, vs. WT mice post-STZ-treatment.

To elucidate the molecular mechanisms underlying the attenuated hyperglycemia in ApoA-IV-Tg mice, we analyzed circulating glucagon levels and hepatic gluconeogenic signaling. Following STZ injection, WT mice exhibited a significant increase in plasma glucagon levels, a response that was markedly blunted in ApoA-IV-Tg mice (Figure 7A). Consistent with elevated glucagon levels, WT mice showed a significant induction of hepatic pCREB/CREB ratio after STZ treatment, whereas this activation was significantly attenuated in the ApoA-IV-Tg group (Figure 7B).

Furthermore, we examined the expression of key gluconeogenic enzymes in the liver. STZ treatment induced a robust increase in G6Pase mRNA expression in WT mice (*p* < 0.01 vs. non-STZ treatment); however, this induction was significantly reduced in ApoA-IV-Tg mice (*p* < 0.05 vs. WT-STZ treatment; Figure 7C). A similar trend was observed for PEPCK mRNA expression; however, the lower transcript levels in ApoA-IV-Tg mice compared to STZ-treated WT controls did not reach statistical significance (Figure 7D). G6pase and PEPCK are key hepatic gluconeogenic enzymes [17]. Together, these findings suggest that ApoA-IV overexpression mitigates hyperglycemia by suppressing glucagon-mediated CREB signaling pathway and subsequent hepatic glucose production.

### 3.8. ApoA-IV Overexpression Blunts the STZ-Induced Surge in Corticosterone and Lipolysis

**Figure 8:**
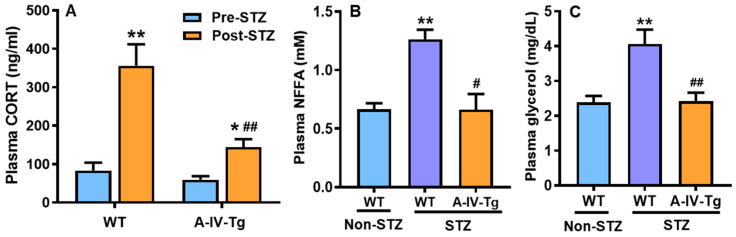
ApoA-IV overexpression suppresses the counter-regulatory stress response and systemic lipolysis following β-cell ablation. (**A**) Plasma corticosterone (CORT) levels in WT and ApoA-IV-Tg mice under basal conditions and following STZ-induced β-cell ablation. (**B**,**C**) Fasting plasma concentrations of non-esterified fatty acids (NEFA; B) and glycerol (**C**) in Non-STZ WT and STZ-treated WT and ApoA-IV-Tg mice. Data are expressed as mean ± SEM (*n* = 6–7 per group). * *p* < 0.05; ** *p* < 0.01, vs. WT mice without STZ-treatment. ^#^ *p* < 0.05; ^##^ *p* < 0.01, vs. WT mice post-STZ-treatment.

To further explore the metabolic benefits of ApoA-IV, we measured plasma markers of the stress response and systemic lipolysis following STZ-induced β-cell ablation. WT mice showed a robust and significant increase in plasma corticosterone (CORT) levels following STZ administration (Figure 8A). Remarkably, this stress-induced surge was significantly blunted in ApoA-IV-Tg mice (*p* < 0.01 vs. WT-STZ), although a modest increase relative to their pre-STZ baseline remained (*p* < 0.05).

Since elevated CORT and glucagon can synergistically drive lipolysis in adipose tissue, we subsequently analyzed circulating lipid metabolites. STZ treatment in WT mice resulted in a significant elevation of plasma non-esterified fatty acids (NEFA) (Figure 8B) and plasma glycerol (Figure 8C), both of which are indicative of accelerated lipolytic activity. In contrast, ApoA-IV-Tg mice were significantly protected against these increases, maintaining NEFA and glycerol levels comparable to those of non-diabetic WT controls. These results suggest that the glycemic protection afforded by ApoA-IV may be partly due to its ability to suppress the counter-regulatory stress response and the subsequent liberation of lipolytic substrates, which otherwise exacerbate hyperglycemia [18].

### 3.9. ApoA-IV Directly Inhibits Glucagon Secretion via LRP1 in Pancreatic Islets

While these in vivo findings demonstrated that ApoA-IV overexpression effectively blunts the post-STZ glucagon surge and suppresses downstream hepatic gluconeogenesis, they did not distinguish whether this effect was mediated indirectly through systemic metabolic improvements or via a direct action on the pancreas. To evaluate the direct cellular function of ApoA-IV on the endocrine pancreas, we next transitioned to an ex vivo approach using primary isolated mouse islet cultures to measure low-glucose-stimulated glucagon secretion.

**Figure 9:**
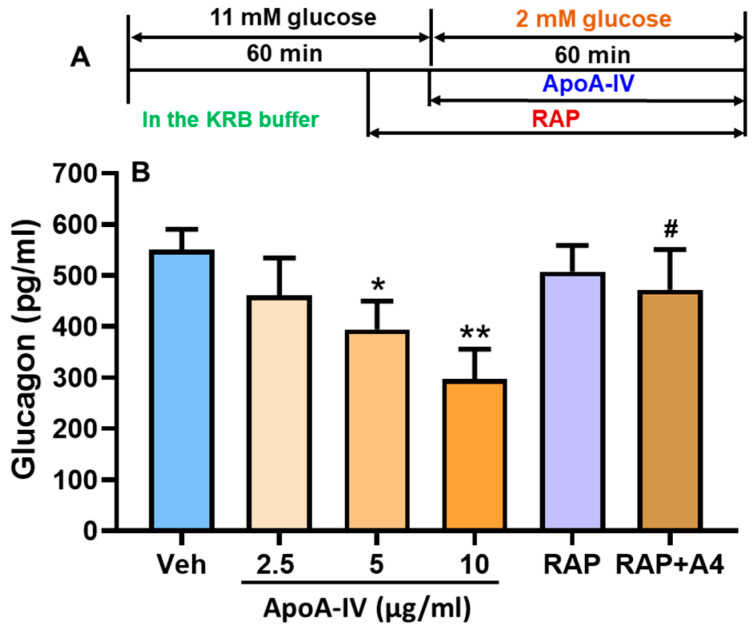
ApoA-IV suppresses glucagon secretion via LRP1 in cultured mouse islets. (**A**) Experimental design for glucagon secretion in isolated islets. Pancreatic islets were pre-incubated in KRB buffer containing 11 mM glucose for 60 min. To stimulate glucagon secretion, islets were incubated in a low-glucose medium (2 mM) for an additional 60 min. Treatments included ApoA-IV at varying concentrations (2.5, 5, and 10 μg/mL). To determine the role of LRP1, the inhibitor Receptor-Associated Protein (RAP, 0.3 μM) was added to the media 15 min prior to the low-glucose challenge. (**B**) Glucagon secretion levels. Low glucose-induced glucagon secretion was significantly suppressed by ApoA-IV in a dose-dependent manner. The inhibitory effect of ApoA-IV (10 μg/mL) was largely abolished by the addition of RAP (RAP + A4), indicating that ApoA-IV acts via the LRP1 receptor to attenuate glucagon release from alpha cells. Data are expressed as mean ± SEM (*n* = 4 independent experiments). * *p* < 0.05; ** *p* < 0.01, vs. vehicle treatment; ^#^ *p* < 0.05, vs. 10 μg/mL ApoA-IV treatment.

We treated isolated primary mouse islets with recombinant ApoA-IV [19]. ApoA-IV significantly inhibited glucagon secretion into the culture medium in a dose-dependent manner (Figure 9B). To identify the receptor mediating this effect, we utilized Receptor-Associated Protein (RAP), a known antagonist of LRP1. While ApoA-IV (10 μg/mL) alone significantly reduced glucagon levels, the addition of RAP largely abolished this inhibitory effect, restoring glucagon concentrations to levels comparable to the control group. These results indicate that ApoA-IV directly suppresses glucagon secretion in pancreatic islets through an LRP1-dependent signaling pathway. This mechanism provides a cellular basis for the attenuated hyperglucagonemia and subsequent reduction in hepatic gluconeogenesis observed in the ApoA-IV-Tg mice.

### 3.10. Co-Localization of LRP1 and Glucagon in Pancreatic Islets

**Figure 10:**
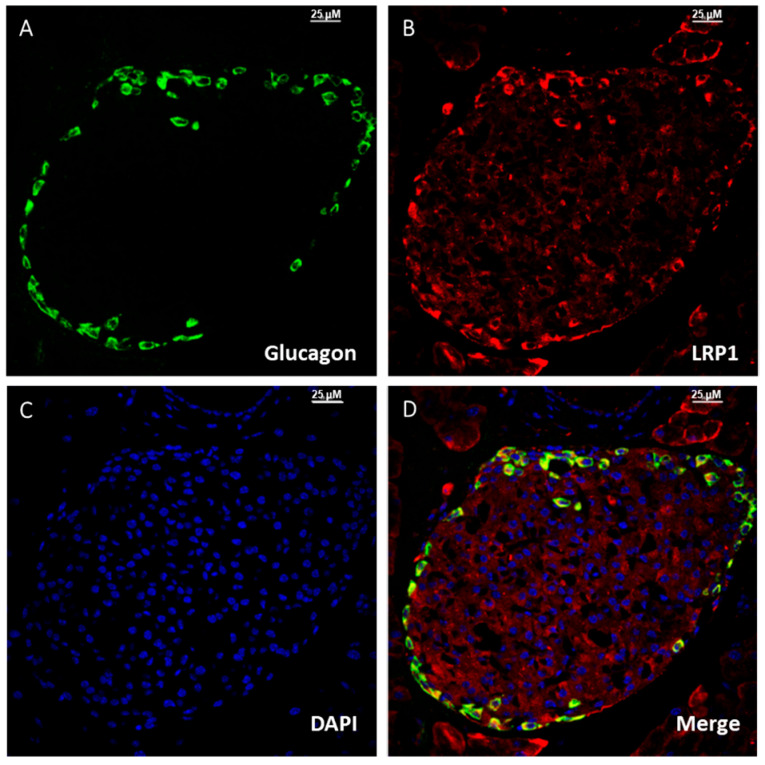
Representative confocal images of a single mouse pancreatic islet processed for triple-fluorescence immunohistochemistry. Glucagon (green fluorescence; (**A**)) marks α cells, while LRP1 (red fluorescence; (**B**)) is shown throughout the islet. Panel (**C**) shows DAPI staining (blue fluorescence) of all nuclei. Panel (**D**) (Merge) is the overlay of glucagon, LRP1, and DAPI signals. Scale bars are 25 μm.

The finding that the LRP1 antagonist RAP completely abolishes ApoA-IV-mediated glucagon suppression functionalizes LRP1 as the obligate receptor driving this cellular response. However, confirming this mechanism required anatomical evidence that the receptor is physically present on the target cells. Therefore, to establish the precise molecular localization underpinning this cellular function, we performed triple-fluorescence immunohistochemistry to examine the spatial distribution and co-localization of LRP1 specifically within the pancreatic α-cell population. As shown in Figure 10, α cells were identified by green fluorescence for glucagon (Figure 10A), while nuclei were visualized via DAPI staining (Figure 10C). LRP1 signals, recognized as red fluorescence (Figure 10B), were observed throughout the islet. Importantly, the merged images (Figure 10D) revealed clear co-localization of LRP1 and glucagon, confirming LRP1’s presence within the α cell population. These findings provide a structural framework for LRP1-mediated ApoA-IV signaling in the regulation of glucagon secretion.

## 4. Discussion

The global escalation of obesity and type 2 diabetes necessitates the identification of endogenous factors capable of simultaneously addressing energy imbalance and glycemic dysregulation [20]. In the present study, we demonstrate that ApoA-IV acts as a potent multi-organ metabolic regulator. By employing a 20-week HFD challenge and a STZ-induced model of β-cell ablation, we reveal that ApoA-IV provides a “dual-defense” mechanism: it accelerates lipid utilization via increased energy expenditure and directly suppresses the α-cell-glucagon axis through an LRP1-dependent pathway.

### 4.1. ApoA-IV Promotes Metabolic Flexibility and Thermogenic Flux

A defining characteristic of the ApoA-IV-Tg phenotype in our study is the remarkable resistance to diet-induced adiposity despite identical caloric intake to WT controls. This “energy-burning” profile is underpinned by a significant elevation in energy expenditure (HEAT) and a consistently lower respiratory exchange ratio (RER). These data suggest that ApoA-IV facilitates metabolic flexibility, allowing the organism to maintain high rates of fatty acid oxidation even under the pressure of chronic lipid oversupply. This is consistent with our previous work demonstrating that ApoA-IV is a critical mediator of diet-induced thermogenesis [21]. Specifically, intestinal ApoA-IV overexpression enhances thermogenesis in brown adipose tissue and elevates energy expenditure [21], whereas ApoA-IV deficiency results in reduced thermogenesis in brown adipose tissue and decreased energy expenditure following HFD consumption [22]. These prior findings reinforce the current observation that ApoA-IV overexpression actively drives a thermogenic program to counteract lipid accumulation.

We note that ApoA-IV-Tg mice entered the HFD challenge with a significantly lower baseline body weight compared to their wild-type counterparts. While such initial baseline variations can sometimes confound long-term phenotypic interpretations, our holistic metabolic data demonstrate that the transgenic mice are actively protected by fundamental physiological shifts, as evidenced by their significantly lower total weight gain and suppressed fat mass accumulation over the 20-week period. The lower adiposity observed over 20 weeks is clearly underpinned by a sustained elevation in thermogenic flux (HEAT) and an enhanced capacity for lipid utilization (lower RER), which persist independently of their starting weight. Thus, the lean phenotype is a consequence of continuous metabolic flexibility and elevated energy expenditure rather than a passive reflection of baseline divergence.

### 4.2. Sustained Preservation of the Insulin-Glucose Homeostatic Set-Point

Beyond body weight management, ApoA-IV significantly improved systemic glycemic control throughout the 20-week HFD challenge. While many metabolic interventions show waning efficacy over time [23], the insulin-sensitizing effects of ApoA-IV remained remarkably robust despite five months of chronic overnutrition. ApoA-IV-Tg mice consistently exhibited lower fasting glucose and insulin levels, culminating in a drastically reduced cumulative HOMA-IR burden and integrated glucose AUC. These results align with previous studies suggesting that ApoA-IV improves glucose uptake in peripheral tissues, such as skeletal muscle and adipose tissue [24]. However, our study significantly extends these findings by demonstrating that these benefits are durable even under the stress of long-term lipid oversupply.

It is worth noting that while the protective effect of ApoA-IV on fasting blood glucose was highly pronounced during the first 12 weeks of the high-fat diet, the statistical difference between genotypes narrowed toward the end of the 20-week challenge (Figure 4A). This flattening effect suggests that extreme, long-term overnutrition may eventually challenge the fasting euglycemic baseline. However, the overall protective role of ApoA-IV remains evident in the context of systemic homeostasis, as evidenced by a significantly reduced cumulative HOMA-IR burden and superior glucose clearance during the 16-week IPGTT. The shifting kinetics of fasting glucose during ultra-long-term dietary challenges represent a nuanced metabolic characteristic that requires further formal observation in future long-term longitudinal studies.

Notably, the improved glucose clearance during IPGTTs in transgenic mice occurred despite significantly lower insulin secretion, a hallmark of enhanced peripheral insulin sensitivity. This finding suggests a profound clinical advantage: by increasing glucose disposal efficiency, ApoA-IV reduces systemic insulin requirements, thereby shielding pancreatic β-cells from the exhaustive secretory demand and eventual failure typically triggered by chronic HFD feeding [25].

### 4.3. A Novel Paradigm: Insulin-Independent Glycemic Protection via α-Cell Suppression

While traditional diabetes management has remained overwhelmingly “insulin-centric,” glycemic homeostasis is fundamentally dictated by the delicate counter-regulatory balance between insulin and glucagon [4,26]. Historically, the therapeutic potential of targeting the α-cell has been underappreciated. However, emerging evidence suggests that hyperglucagonemia, rather than insulin deficiency alone, may be the sine qua non of diabetic pathology [4,27]. Indeed, glucagon excess is a hallmark of all forms of poorly controlled diabetes, and genetic ablation of glucagon receptors has been shown to protect mice from hyperglycemia even after total β-cell loss [28].

The most clinically provocative finding of this study is the profound glycemic resilience afforded by ApoA-IV in the near-total absence of insulin. In our STZ model, a gold standard for GLUT2-mediated β-cell alkylation and catastrophic death [29], ApoA-IV-Tg mice remained strikingly resistant to overt hyperglycemia despite near-undetectable insulin levels. This dramatic disconnect between systemic insulin concentrations and glucose excursions necessitates a fundamental shift in focus toward the historically “forgotten” half of the bi-hormonal hypothesis [4].

Mechanistically, glucagon targets the liver to activate the cyclic AMP (cAMP)/protein kinase A (PKA) signaling cascade, which phosphorylates CREB to drive the expression of rate-limiting gluconeogenic genes, specifically G6pc (G6Pase) and Pck1 (PEPCK) [30,31]. In the present study, while basal glucagon levels were comparable between genotypes, the post-STZ glucagon “surge” was markedly blunted in ApoA-IV-Tg mice. This attenuation of hyperglucagonemia directly correlated with suppressed hepatic CREB phosphorylation and reduced G6pc expression. By acting as a molecular “brake” on the α-cell, ApoA-IV effectively “clamps” hepatic glucose production at its source.

Interestingly, while post-STZ hepatic G6Pase mRNA expression was significantly blunted in ApoA-IV-Tg mice, the down-regulation of PEPCK did not reach statistical significance. This divergence likely reflects the distinct regulatory architectures of these enzymes; unlike G6Pase, PEPCK is highly sensitive to additional inputs, such as cellular energy charge and fatty acid concentrations. This distinction is critical when interpreting other systemic changes: the observed suppression of the post-STZ corticosterone surge and peripheral lipolysis should not be definitively viewed as independent upstream drivers of PEPCK expression or glycemic stability. Instead, these lipolytic and hormonal reductions are likely secondary to the primary suppression of hyperglucagonemia, representing a coordinated metabolic buffer rather than a direct cause.

Our previous ‘loss-of-function’ studies established that ApoA-IV knockout (KO) mice exhibit significant glucose intolerance that can be rescued by exogenous ApoA-IV administration [7,24]. Conversely, the current ‘gain-of-function’ study reveals that ApoA-IV-Tg mice maintain euglycemia with a significantly reduced insulin requirement. While these data demonstrate a broad-spectrum antidiabetic action, the potential contribution of residual insulin action post-STZ warrants consideration. Although plasma insulin levels dropped to nearly undetectable levels, trace amounts of circulating hormone cannot be entirely excluded in vivo. Because ApoA-IV actively enhances peripheral insulin sensitivity, increased sensitivity to this small residual pool of insulin may synergize with our newly uncovered hyperglucagonemia suppression pathway to blunt overt hyperglycemia. Thus, while ApoA-IV plays a critical role in maintaining the glycemic ‘set-point’ during severe insulin deficiency, a complementary component of enhanced insulin sensitivity may contribute to the overall phenotype, positioning it as a promising candidate for further investigation as an adjunct therapy to reduce exogenous insulin dependency.

### 4.4. The LRP1-ApoA-IV Axis: A Molecular Bridge in the Islet

We have identified LRP1 as a novel receptor in adipose tissues [9]. Mechanistically, we provide the first evidence that this α-cell suppression is a direct, receptor-mediated event. Our in vitro results demonstrate that recombinant ApoA-IV directly inhibits glucagon secretion from primary islets in a dose-dependent manner. This inhibitory effect of ApoA-IV was abolished by the LRP1 antagonist, receptor-associated protein (RAP) [32]. Furthermore, LRP1 co-localized with glucagon in pancreatic α-cells, as confirmed by immunofluorescence microscopy. Collectively, these findings identify LRP1 as a critical mediator of ApoA-IV signaling and its subsequent regulation of glucagon secretion within the islet.

### 4.5. Buffering the “Metabolic Storm”: Suppression of Stress and Lipolysis

Beyond its direct effects on the α-cell, ApoA-IV appears to mitigate the systemic “metabolic storm” typically triggered by acute insulin deficiency. In our WT cohort, STZ administration induced a robust surge in plasma corticosterone and a subsequent explosion of systemic lipolysis, evidenced by sharply elevated NEFA and glycerol. This hormonal and metabolic shift creates a vicious cycle; insulin deficiency in rodents is known to accelerate hepatic gluconeogenesis in part through CORT-driven adipocyte lipolysis [33,34].

The liberation of glycerol provides a direct substrate for glucose synthesis, while the influx of free fatty acids (FFAs) increases hepatic acetyl-CoA concentrations, a potent allosteric activator of the rate-limiting gluconeogenic enzyme, pyruvate carboxylase [35,36]. In striking contrast, ApoA-IV-Tg mice exhibited a significantly attenuated stress response following STZ treatment. By buffering the rise in corticosterone and stabilizing peripheral lipid mobilization, ApoA-IV prevents the liver from being flooded with gluconeogenic precursors and metabolic activators. This unique ability to suppress the counter-regulatory stress-lipolysis axis further reinforces the glycemic protective role of ApoA-IV and distinguishes it from traditional incretin-based therapies, which primarily focus on insulin and glucagon secretion rather than systemic substrate flux [37,38].

### 4.6. Rethinking the Bi-Hormonal Hypothesis and Therapeutic Potential

For decades, the management of diabetes has been dominated by an “insulin-centric” view [39,40]. However, our results provide compelling evidence for the “bi-hormonal hypothesis,” which depicts the symptoms of diabetes as being as much a result of glucagon excess as they are of insulin deficiency. The fact that ApoA-IV-Tg mice remain euglycemic even after severe β-cell loss highlights a critical therapeutic window: if glucagon can be sufficiently suppressed, the requirement for exogenous insulin may be significantly reduced.

The dual-action nature of ApoA-IV, which addresses both lipid-driven energy expenditure in the periphery and hormone-driven glucose production in the liver, distinguishes it from current GLP-1 receptor agonists. While GLP-1 also inhibits glucagon [41], ApoA-IV’s ability to stabilize peripheral lipid mobilization and counter the corticosterone surge suggests a broader “metabolic buffer” effect that could be particularly beneficial in cases of poorly controlled diabetes or metabolic crises.

### 4.7. Limitations and Future Directions

While the metabolic protection observed in this study is robust, certain characteristics of our experimental models warrant consideration. First, because our transgenic model overexpresses ApoA-IV specifically within the intestine, resulting in elevated systemic levels, it remains difficult to fully distinguish the endocrine effects of circulating ApoA-IV from potential local factors within the pancreatic islets. Second, while our in vitro data strongly support a receptor-mediated mechanism, the current study does not include an α-cell-specific LRP1 knockout mouse line to provide a corresponding in vivo genetic verification.

While the precise post-receptor molecular intermediates within α-cells remain uncharacterized, several potential intracellular pathways downstream of LRP1 warrant consideration. LRP1 possesses a versatile cytoplasmic domain containing NPxY motifs that serve as prominent docking sites for scaffolding and signaling adaptors. In the context of the pancreatic α-cell, ApoA-IV-mediated LRP1 activation might suppress glucagon secretion by directly inhibiting adenylate cyclase activity, thereby lowering intracellular cyclic AMP (cAMP) levels and dampening protein kinase A (PKA) signaling. Alternatively, LRP1 activation has been shown in other cell types to modulate intracellular calcium (Ca^2+^) handling or recruit survival and metabolic kinase networks, such as the phosphoinositide 3-kinase (PI3K)/Akt and mitogen-activated protein kinase (MAPK) cascades. Delineating which of these specific secondary messenger systems coupled to LRP1 are engaged by ApoA-IV represents an important mechanistic focus for our future investigation.

Additionally, the acute STZ model primarily simulates the severe β-cell loss and insulin deficiency characteristic of Type 1 diabetes. Caution should be exercised when extrapolating these mechanisms to Type 2 diabetes and metabolic syndrome, where insulin resistance and hyperinsulinemia predominate. Finally, significant clinical translational challenges remain. Important species-specific differences in apolipoprotein biology, cellular distribution, and vascular architecture exist between rodent and human islets. Extensive future verification in human islet tissue and non-rodent models will be necessary to determine the clinical utility of this insulin-independent axis.

## 5. Conclusions

In summary, our study establishes ApoA-IV as a sophisticated multi-organ metabolic regulator that effectively coordinates energy balance and glycemic control. By maintaining elevated metabolic rates and promoting lipid oxidation, ApoA-IV significantly attenuates diet-induced obesity. Simultaneously, it preserves glucose homeostasis by directly modulating the pancreatic α-cell/LRP1 axis, thereby suppressing hyperglucagonemia and the subsequent induction of hepatic gluconeogenesis. As the global prevalence of severe insulin resistance and metabolic syndrome continues to rise, these findings position the ApoA-IV-LRP1 signaling pathway as a highly promising target for the development of next-generation, dual-action metabolic therapies designed to treat the interconnected pathologies of obesity and diabetes.

## Data Availability

The original contributions presented in this study are included in the article/Supplementary Materials. Further inquiries can be directed to the corresponding author.

## Supplementary Materials

The following supporting information can be downloaded at: https://www.mdpi.com/article/10.3390/cells15131229/s1, Figure S1: Impact of ApoA-IV overexpression on adiposity and liver weight in WT and ApoA-IV-Tg mice. (A) Visceral fat, (B) subcutaneous fat, (C) interscapular brown adipose tissue (BAT), and (D) liver weight. All tissue weights are expressed as a percentage of total body weight. Figure S2: Longitudinal respiratory gas exchange measured by indirect calorimetry at Week 17 of HFD feeding. Top Panel: Oxygen consumption (VO_2_): ApoA-IV-Tg mice exhibited consistently higher levels than WT controls throughout the 24-h cycle. Bottom Panel: Carbon dioxide production (VCO_2_): Similar to oxygen consumption, it was elevated in ApoA-IV-Tg mice relative to WT mice, particularly at the onset of the dark phase. Dark/Light Cycles: For calorimetry, white and black bars on the x-axis represent the light and dark cycles, respectively. Figure S3: Locomotor activity of WT and ApoA-IV-Tg mice during HFD feeding. Locomotor activity was assessed at week 17 of HFD feeding using a multi-beam infrared monitoring system to determine whether differences in energy expenditure were associated with physical movement. (A) Cumulative Activity: Total beam-break counts recorded over a 24-h period showed no significant difference in total physical activity between WT and ApoA-IV-Tg mice. (B) Average hourly light-on and (C) light-off activities: Mean activity levels during the 12-h light and dark cycles were compared across genotypes. No statistically significant divergence was observed, indicating that the elevated dark-phase HEAT observed in Figure 2 is independent of changes in locomotion. Figure S4: Effect of ApoA-IV overexpression on body weight and food intake following STZ-induced β-cell ablation. A–B: Longitudinal monitoring of body weight (A) and daily food intake (B) in WT and ApoA-IV-Tg mice during the 5-day period following STZ injection. While A-IV-Tg mice exhibited lower baseline body weights than WT controls, both genotypes showed a similar pattern of weight loss after STZ induction. No significant differences in daily food consumption were observed between genotypes (B), indicating that the glycemic protection observed in A-IV-Tg mice is independent of changes in caloric intake.

## Abbreviations

ApoA-IV	Apolipoprotein A-IV
AUC	Area under the curve
BAT	Brown adipose tissue
CHOL	Cholesterol
CORT	Corticosterone
EE	Energy expenditure
G6pase	Glucose-6-phosphatase
GSGS	Glucose-stimulated glucagon secretion
HFD	High-fat diet
HOMA-IR	Homeostatic model assessment of insulin resistance
IPGTT	Intraperitoneal glucose tolerance tests
LRP1	Low-density lipoprotein receptor-related protein 1
NEFA	Non-esterified fatty acids
pCREB	Phosphorylated cAMP-response-element-binding protein
PEPCK	Phosphoenolpyruvate carboxykinase
PKA	cAMP)/protein kinase A
RAP	Receptor-associated protein
RER	Respiratory exchange ratio
STZ	Streptozotocin
TG	Triglycerides cholesterol
Total CREB	Total cAMP-response-element-binding protein
VO_2_	Oxygen consumption
VCO_2_	Carbon dioxide production
