# Supplementary material for: The ApoA-IV–LRP1 Signaling Axis: A Novel Insulin-Independent Pathway for the Suppression of Diabetic Hyperglucagonemia
Source: Cells. 2026 Jul 7;15(13):1229. doi: 10.3390/cells15131229 (PMC13359458; doi:10.3390/cells15131229)
Supplement: Supplementary file 1 [file cells-15-01229-s001.zip › cells-4379440-supplementary.pdf]

Article

The ApoA-IV–LRP1 Signaling Axis: A Novel Insulin-Independent Pathway for the Suppression of Diabetic Hyperglucagonemia.

Supplementary data S1:

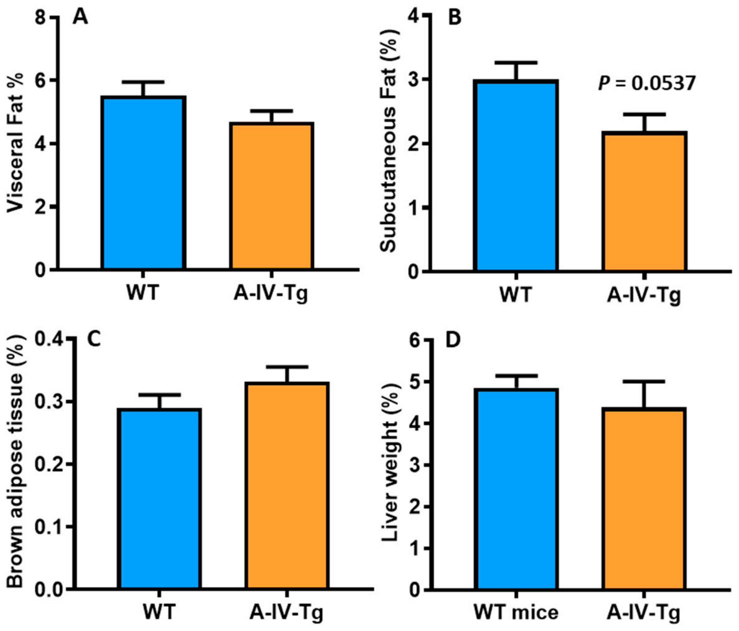

Supplementary Figure S1. Impact of ApoA-IV overexpression on adiposity and liver weight in WT and ApoA-IV-Tg mice. (A) Visceral fat, (B) subcutaneous fat, (C) interscapular brown adipose tissue (BAT), and (D) liver weight. All tissue weights are expressed as a percentage of total body weight. Data are presented as mean ± SEM (n = 5-7 per group).

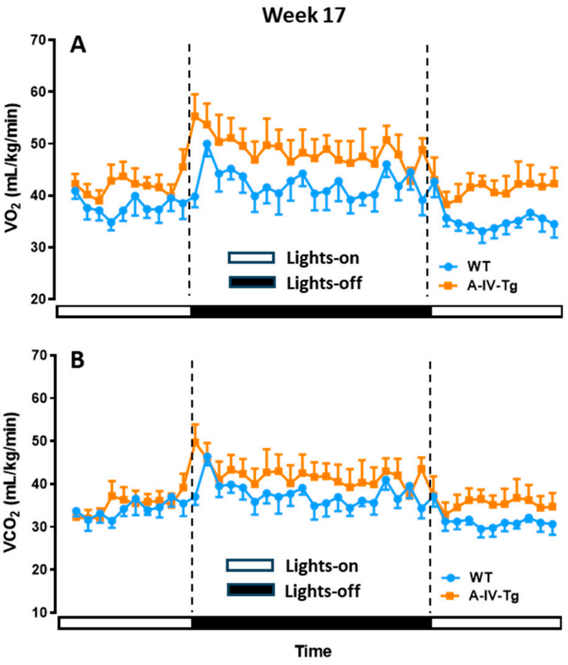

Supplementary Figure S2. Longitudinal respiratory gas exchange measured by indirect calorimetry at Week 17 of HFD feeding. Top Panel: Oxygen consumption (VO_2): ApoA-IV-Tg mice exhibited consistently higher levels than WT controls throughout the 24-hour cycle. Bottom Panel: Carbon dioxide production (VCO_2): Similar to oxygen consumption, it was elevated in ApoA-IV-Tg mice relative to WT mice, particularly at the onset of the dark phase. Dark/Light Cycles: For calorimetry, white and black bars on the x-axis represent the light and dark cycles, respectively. Data are presented as mean ± SEM (n = 5-6 per group).

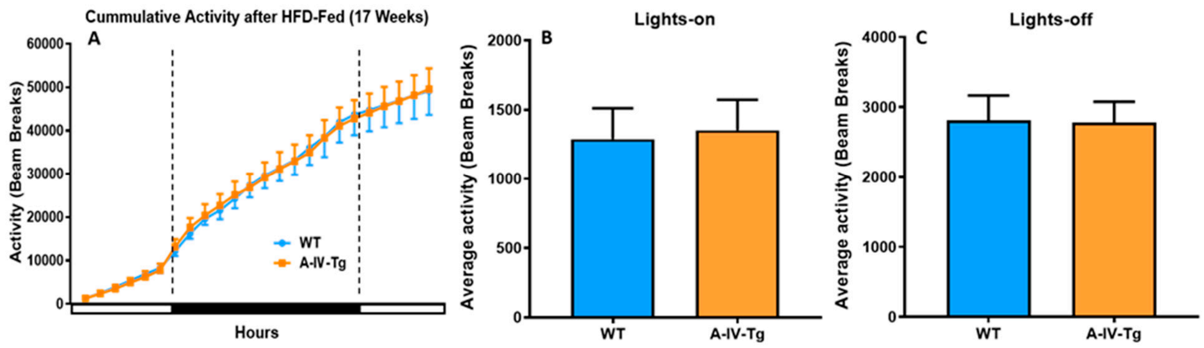

Supplementary Figure S3: Locomotor activity of WT and ApoA-IV-Tg mice during HFD feeding. Locomotor activity was assessed at week 17 of HFD feeding using a multi-beam infrared monitoring system to determine whether differences in energy expenditure were associated with physical movement. (A) Cumulative Activity: Total beam-break counts recorded over a 24-hour period showed no significant difference in total physical activity between WT and ApoA-IV-Tg mice. (B) Average hourly light-on and (C) light-off activities: Mean activity levels during the 12-hour light and dark cycles were compared across genotypes. No statistically significant divergence was observed, indicating that the elevated dark-phase HEAT observed in Figure 2 is independent of changes in locomotion. Data are presented as mean ± SEM (n = 5-6 per group).

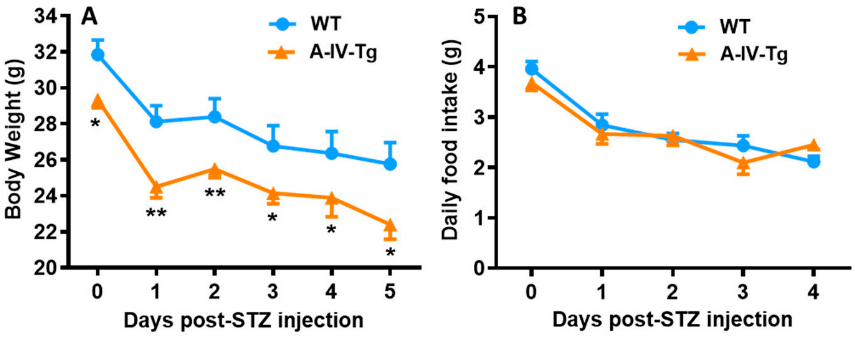

Supplementary Figure S4: Effect of ApoA-IV overexpression on body weight and food intake following STZ-induced β-cell ablation. A–B: Longitudinal monitoring of body weight (A) and daily food intake (B) in WT and ApoA-IV-Tg mice during the 5-day period following STZ injection. While A-IV-Tg mice exhibited lower baseline body weights than WT controls, both genotypes showed a similar pattern of weight loss after STZ induction. No significant differences in daily food consumption were observed between genotypes (B), indicating that the glycemic protection observed in A-IV-Tg mice is independent of changes in caloric intake. Data are presented as mean ± SEM (n = 6-7 per group). * $p < 0.05$, ** $p < 0.01$, vs. WT within the same time point.
